# Neurophysiology-Driven Parameter Selection in nTMS-Based DTI Tractography: A Multidimensional Mathematical Model

**DOI:** 10.3389/fnins.2019.01373

**Published:** 2019-12-18

**Authors:** Kathrin Machetanz, Leonidas Trakolis, Maria Teresa Leão, Marina Liebsch, Kristin Mounts, Benjamin Bender, Ulrike Ernemann, Alireza Gharabaghi, Marcos Tatagiba, Georgios Naros

**Affiliations:** ^1^Department of Neurosurgery, Eberhard Karls Universität Tübingen, Tübingen, Germany; ^2^Department of Neuroradiology, Eberhard Karls Universität Tübingen, Tübingen, Germany

**Keywords:** diffusion tensor imaging, fiber tracking, navigated transcranial magnetic stimulation, fractional anisotropy threshold, fiber length threshold, corticospinal tract

## Abstract

**Object:** There is an increasing interest in preoperative diffusion tensor imaging-based fiber tracking (DTI-FT) to preserve function during surgeries in motor eloquent brain regions. However, DTI tractography is challenged by inherent presumptions during particular tracking steps [e.g., deterministic vs. probabilistic DTI, fractional anisotropy (FA) and fiber length (FL) thresholding] and the missing “ground truth” information. In the present study, we intended to establish an objective, neurophysiology-driven approach for parameter selection during DTI-FT of the corticospinal tract integrating both imaging and neurophysiological information.

**Methods:** In ten patients with lesions in eloquent motor areas, preoperative navigated transcranial magnetic stimulation (nTMS) was performed, followed by individual deterministic DTI-FT from a grid of cortical seed points. We investigated over 300 combinations of FA and FL thresholds and applied subsequently a multidimensional mathematical modeling of this empirical data. Optimal DTI parameters were determined by the relationship between DTI-FT (i.e., number of fibers, NoF) and nTMS (i.e., amplitudes of motor-evoked potentials) results. Finally, neurophysiological DTI parameters and the resulting tractography were compared to the current standard approaches of deterministic DTI fiber tracking with a 75% and 50% FA and a FL threshold of 110 mm as well as with intraoperative direct cortical and subcortical stimulation.

**Results:** There was a good goodness-of-fit for the mathematical model (*r*^2^ = 0.68 ± 0 13; range: 0.59–0.97; *n* = 8) except of two cases. Neurophysiology-driven parameter selection showed a high correlation between DTI-FT and nTMS results (*r* = 0.73 ± 0.16; range: 0.38–0.93). In comparison to the standard approach, the mathematically calculated thresholds resulted in a higher NoF in 75% of patients. In 50% of patients this approach helped to clarify the exact tract location or to detect additional functional tracts, which were not identified by the standard approach. This was confirmed by direct cortical or subcortical stimulation.

**Conclusion:** The present study evaluates a novel user-independent method to extract objective DTI-FT parameters that were completely based on neurophysiological data. The findings suggest that this method may improve the specificity and sensitivity of DTI-FT and thereby overcome the disadvantages of current approaches.

## Introduction

In contemporary brain tumor surgery, it is essential to obtain a comprehensive extend of resection while simultaneously preserving functionally relevant structures (e.g., the CST). The former has an important impact on survival, especially in cases of malignant brain lesions (e.g., gliomas), while the latter preserves patients’ quality of life (Jakola et al., 2012; Brown et al., 2016). Thus, there is an increasing interest in functional imaging and mapping methods (e.g., fMRI, MEG, DTI, nTMS) to preoperatively identify eloquent areas (Kamada et al., 2005; Tarapore et al., 2012; Ottenhausen et al., 2015; Hervey-Jumper and Berger, 2016). In this context, DTI-FT has achieved increasing popularity for visualization of specific white matter tracts (Basser et al., 2000; Clark et al., 2003; Nimsky et al., 2006; Assaf and Pasternak, 2008). However, a basic limitation of DTI tractography is the absent “ground truth” information of the displayed fibers (Jbabdi and Johansen-Berg, 2011; Maier-Hein, 2017). Thus, numerous tractography algorithms have been developed to improve each step of the data processing pipeline of DTI-FT: (a) data acquisition (e.g., HARDI instead of DTI), (b) mathematical modeling for fiber reconstruction (e.g., multiple-fiber instead of single-fiber reconstructions), (c) computing the tractography (e.g., probabilistic instead of deterministic methods), and (d) interpretation of fiber tracts (e.g., thresholding on FA and FL values) (Chung et al., 2011; Essayed et al., 2017). Nevertheless, all of these methods may yield false positive or false negative tracts. In addition, these approaches are often time consuming and, therefore, remain challenging to integrate into the neurosurgical routine. In this context, the “simple” deterministic DTI is still the most commonly applied technique which, however, necessitates (a) manual seeding (i.e., selection of ROI) and (b) manual selection of stopping thresholds (e.g., for FA and FL values) (Essayed et al., 2017). However, these manual interventions in the data analysis inherently create uncertainty in the results of deterministic DTI. Accordingly, there have been several attempts to standardize seeding and threshold selection in deterministic DTI. The latest development impacting standard clinic care was the introduction of the nTMS in presurgical DTI-FT, introduced with the intention to standardize ROI seeding (Frey et al., 2012; Krieg et al., 2012; Raffa et al., 2017; Weiss Lucas et al., 2017). In nTMS, a neuro-navigation system is used to define functional cortical spots that elicit electromyographic responses (i.e., MEP) in the contralateral limb following magnetic stimulation. These spots are then used as starting points for the descending fibers. However, this approach leaves the thresholding problem unresolved. While some studies postulated using standardized FA and FL values (e.g., 75% of the individual FA and a FL threshold of 110 mm) (Frey et al., 2012), other studies recommend individual thresholds (Weiss Lucas et al., 2017). However, this is of particular importance as the threshold selection has a tremendous impact on the number of detected fibers. Low FA/FL thresholds increase the number of false positive connections, while high FA/FL thresholds result in an increased number of false negative connections.

In the present study, we suggest an advancement of the nTMS-based DTI-FT toward a purely neurophysiology-driven threshold selection. We conjecture that the current practice misses additional nTMS information beyond the mere *starting point* of descending fibers. By measuring the amplitude of the MEP, nTMS offers an additional measure for the *strength* of the corticospinal connection. We hypothesize a positive relationship between the NoF detected by the DTI-FT and the MEP amplitude measured with nTMS. Such a finding would justify a novel approach; specifically, selecting DTI thresholds in a way to maximize this relationship.

The aim of the present study was to develop and investigate a completely neurophysiology-driven approach for parameter selection during DTI-FT of the CST, integrating both the imaging and neurophysiological information garnered from nTMS mappings.

## Materials and Methods

### Patients

We retrospectively analyzed 10 consecutive patients (52.5 ± 19 years, four female) with motor eloquent lesions who underwent a nTMS in the Neurosurgical Department of the University of Tübingen. The patients were selected according to important neurosurgical indications for presurgical nTMS measurements in motor eloquent lesions (i.e., high-grade gliomas, low-grade gliomas, metastases and vascular lesions). Details of clinical and demographic characteristics of the patients are depicted in Table 1. The study was approved by the local ethics committee of the Eberhard Karls Universität Tübingen.

**TABLE 1:** Characteristics of the patient cohort.

**Patient**	**Sex**	**Age**	**Affected hemisphere**	**Histology**	**Preoperative motor status**
1	m	65	left	Arteriovenous malformation	no paresis
2	m	57	right	Ganglioglioma (WHO I)	no paresis
3	f	69	left	Metastasis (mamma carcinoma)	brachiofacial paresis
4	f	63	right	Glioblastoma (WHO IV)	brachiofacial paresis
5	m	54	right	Glioblastoma (WHO IV)	no paresis
6	m	60	left	Oligodendroglioma (WHO II)	no paresis
7	m	11	right	Ganglioglioma (WHO I)	no paresis
8	f	27	right	Astrocytoma (WHO II)	slight paresis left hand
9	m	49	left	Metastasis (bronchial carcinoma)	no paresis
10	f	70	right	Glioblastoma (WHO IV)	left hemiparesis

### Magnetic Resonance Imaging (MRI)

**FIGURE 1:**
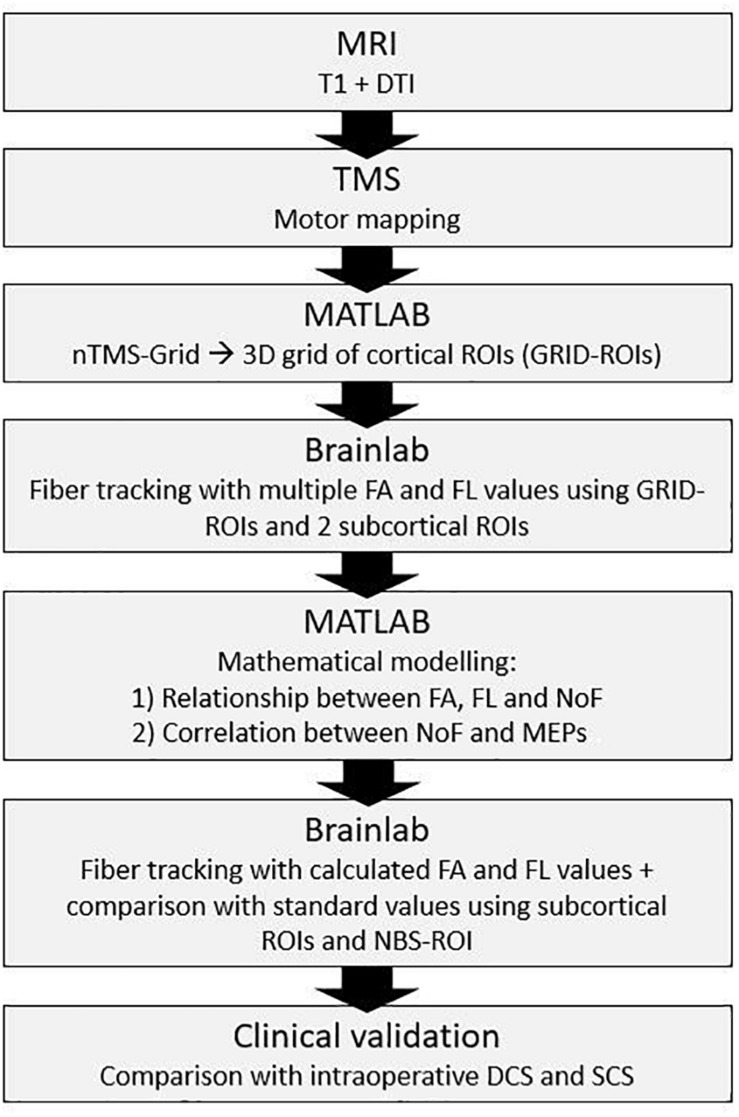
Schematic illustration of the study design.

**FIGURE 2:**
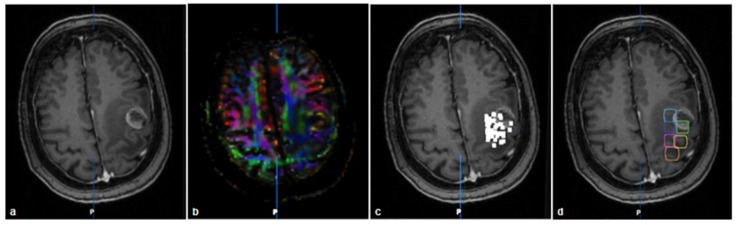
Imaging and grid-building in patient no. 3: T1 with contrast medium **(a)**, FA-map **(b)**, nTMS-map of positive (i.e., amplitude >50 μV) MEP-responses **(c)**, fusion of T1 and GRID-ROI **(d)**.

In all patients, preoperative MR imaging was performed using a 3 or 1.5 T MR imaging unit (Skyra/Prisma-fit/Aera, Siemens Healthineers, Erlangen, Germany) with an 8-channel head coil. Besides conventional imaging including T1-weighted (contrast-enhanced) echo sequences, DTI was performed with a single-shot spin echo at *a b*-value of 1000 s/mm^2^ along 12 to 64 geometric directions. Following, the anatomical MRI data set was imported to our nTMS system (Nexstim Eximia, version 3.2.2, Helsinki, Finland) for further data acquisition and analysis (Figures 1, 2).

### Navigated Transcranial Magnetic Stimulation (nTMS)

We conducted nTMS using the Nexstim Eximia system (version 3.2.2, Helsinki, Finland) with a biphasic figure-of-eight TMS coil. After co-registration of the patient with the anatomical T1-weighted MRI by prominent anatomical landmarks, motor mapping of the lesioned hemisphere was performed. During mapping, the rMT was determined (defined as the stimulus intensity which induces at least 5/10 MEPs > 50 μV) at the EMG surface electrodes of the APB, the FD and/or the EDC muscles contralateral to the lesioned hemisphere by using the integrated EMG unit. Afterward, mapping was conducted at a stimulation intensity of 110% rMT beginning at the hot spot and continuing to the surrounding area as well as the area near to the tumor in a close grid. After manually performing artifact rejection of MEPs displaying atypical latencies, or EMG pre-activation, the nTMS data was exported into a neuro-navigation planning system (BrainLab iPlan cranial 3.0, Brainlab AG, Feldkirchen, Germany) and subsequently into MATLAB (MathWorks, Inc., Natick, MA, United States) using the DICOM standard.

### Cortical ROI Selection and Fiber Tracking

In order to unify the cortical resolution of the individual nTMS examinations and to reduce the spatial dimensionality of the data on the cortical level, we down-sampled the individual nTMS map to a three-dimensional grid with a resolution of 1 × 1 × 1 cm (see Figures 2C,D). For this purpose, we calculated the geometric mean of the DICOM coordinates for the cortical nTMS sweet spot encompassing the five maximal MEP amplitudes. Afterward, preceding away from this hotspot in a horizontal, perpendicular, and diagonal direction, every 1 cm additional cortical ROIs (GRID-ROIs) were fitted in order to cover the entire individual nTMS map. This resulted in 6 to 12 cortical GRID-ROIs for each patient. For each ROI, we calculated the arithmetic mean of all MEP amplitudes evoked within in this area. The modeled grid was imported into the BrainLab iPlan 3.0 software and fused to the original nTMS data, anatomical T1-weigthed MRI, and DTI dataset. In addition to the cortical ROIs, we created two subcortical ROIs for every subject: the first one (PON-ROI) was placed in the caudal pons based on the color-coded FA map (Rosenstock et al., 2017), while the second (PED-ROI) was located below the cerebellar peduncles to avoid aberrant tracts to the cerebellum. Furthermore, for later comparative analysis, we created an additional cortical ROI from the summation of nTMS points of positive MEPs (i.e., amplitude >50 μV), which were enlarged by 2 mm (nTMS-ROI) in line with the current clinical practice (Krieg et al., 2012).

### Mathematical Modeling of the Relationship Between FA and FL Thresholds and the Number of Detected Fibers

**FIGURE 3:**
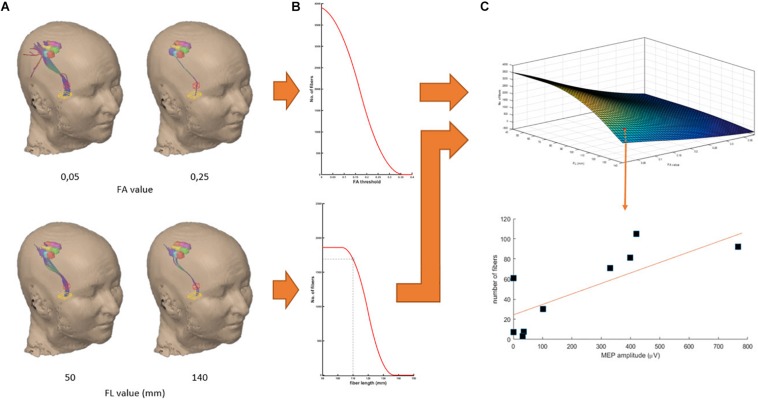
Work-flow demonstrated in the data of patient no. 4. Fitting of the relationship between NoF and FA threshold **(A, top)** and between the NoF and FL threshold **(A, below)** by sigmoidal functions **(B)** and modeling of the three-dimensional relation of NoF, FA, and FL thresholds **(C)**. Finally, for each pair of FA and FL thresholds we calculated the correlation coefficient of the relation between the resulting NoF for each GRID-ROI and the corresponding MEP amplitude.

In order to understand and to enable mathematical modeling of the multivariate relationship between FA and FL thresholds to the NoF, we performed DTI-FT for each GRID-ROI (together with the PON-ROI and PED-ROI) applying over 300 combinations of FA and FL thresholds. Therefore, we successively increased the FL values from 40 to 140 mm (in steps of 20 mm) and evaluated FA values from 0.01 to the 100% FAT (in steps of 0.03). This revealed a sigmoidal relationship between the FA values and NoF as well as between the FL values and NoF (Figure 3). These relationships were then fitted to sigmoidal functions:

(1) $$f\,\left(x\right)=\frac{a}{1+e^{-b\,\left(x-c\right)}}$$

(2) $$g\,\left(y\right)=\frac{d}{1+e^{-j\,\left(y-k\right)}}$$

where f(*x*) represents NoF contingent on different FL thresholds *x*, and g(*y*) represents NoF contingent on different FA thresholds *y. a, b, c, d, j, and k* are constants. Finally, we modeled the 3-dimensional relation between FL and FA thresholds and NoF by

(3) h⁢(x,y)=[(l*f⁢(x))*(m*g⁢(y))]+n

with *l, m, n* being additional constants. This approach resulted in precise fitting results for all except of two patients (Table 2) which were excluded from group analysis.

**TABLE 2:** Individual nTMS-DTI results.

**Patient**	**1**	**2**	**3**	**4**	**5**	**6**	**7**	**8**	**9**	**10**
**Standard of 75% FAT and 110 mm FL**										
75% FAT	0.31	0.28	0.1	0.23	0.28	0.17	0.28	0.34	0.11	0.23
FL thresh.	110	110	110	110	110	110	110	110	110	110
NoF	121	6	11	175	225	41	83	123	805	23
**Standard of 50% FAT and 110 mm FL**										
50% FAT	0.21	0.19	0.07	0.16	0.19	0.11	0.19	0.23	0.08	0.15
FL thresh.	110	110	110	110	110	110	110	110	110	110
NoF	862	1103	123	537	951	112	566	599	1119	97
**Mathematical model**										
R	0.67	0.88	0.83	0.71	0.92	0.93	0.85	0.81	0.70	0.38
R^2^	0.59	0.65	0.59	0.72	0.60	0.97	0.73	0.61	0.03	−0.08
FA thresh.	0.26	0.24	0.18	0.14	0.08	0.31	0.23	0.23	0.05	0.01
FL thresh.	125	135	95	95	170	85	130	105	150	75
NoF	389	107	10	682	264	562	55	725	217	612

### Relationship Between DTI Parameters and MEP Amplitude

**FIGURE 4:**
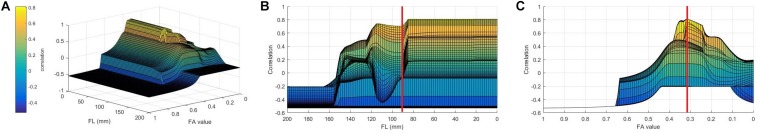
Group results of the multi- **(A)** and two-dimensional interactions **(B,C)** between FA, FL, and the Pearson’s correlation coefficient between the resulting NoF and the MEP amplitudes. The local maximum of FA and FL thresholds maximizing the correlations between resulting NoF and MEP amplitudes represents optimal DTI parameter selection (see red line).

Subsequently, we used the mathematical modeling [3] to re-calculate the expected NoF for a combination of 4000 pairs of FA (0–1 in 0.01 steps) and FL (0–200 mm in 5 mm steps) values for each GRID-ROI. These NoF values were then correlated (Pearson’s correlation) to the mean MEP amplitudes of the GRID-ROIs. This approach resulted in a complex multidimensional relationship with different local maxima (Figure 4). The local maximum of FA and FL thresholds maximizing the correlations between resulting NoF and MEP amplitudes represents optimal DTI parameter selection (see red line in Figure 4).

### Comparative Study

We applied the calculated optimal FA and FL thresholds to the nTMS -ROI (together with the PON-ROI and PED-ROI) within the BrainLab software in accordance with the current clinical standard (Krieg et al., 2012). The resultant NoF of the resulting tract was compared to the standard approach with a 75%- and 50%-FAT and a FL threshold of 110 mm (Frey et al., 2012).

### Intraoperative Evaluation of the DTI-FT

**FIGURE 5:**
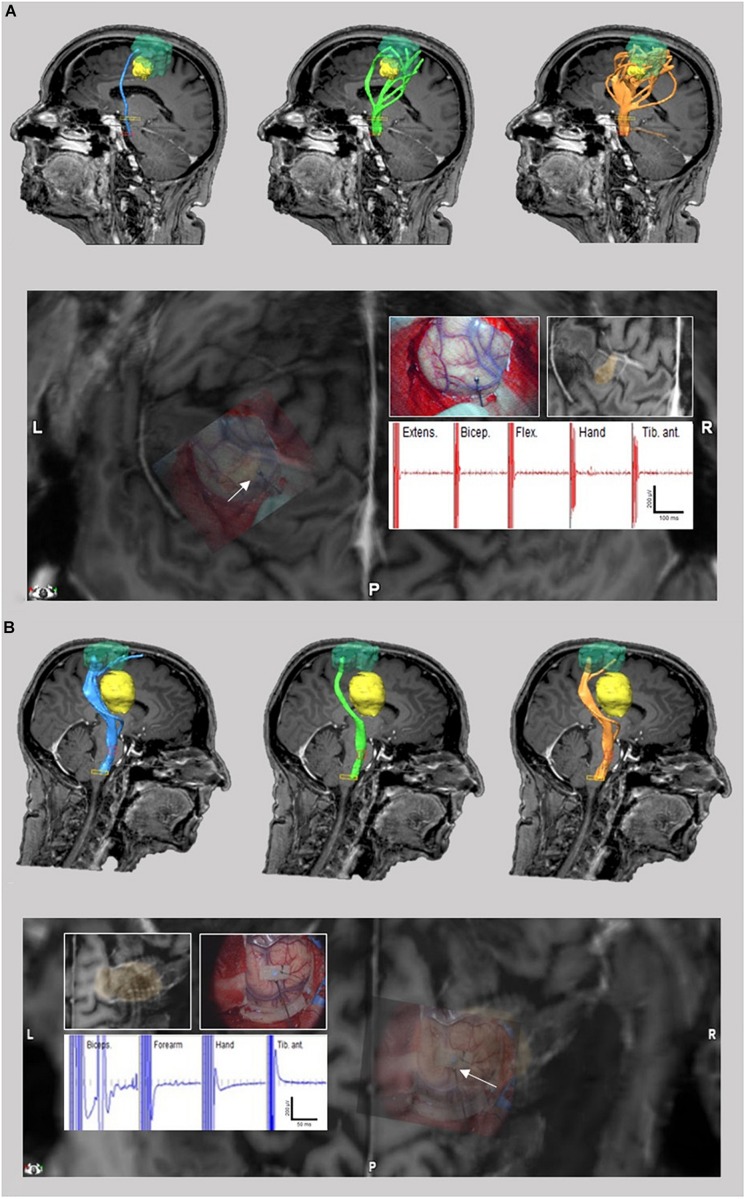
TMS-based DTI fiber tracking of patient no. 3 **(A)** and 4 **(B)**. Comparison between tracts resulting from FA and FL parameter selection with our neurophysiology-driven approach (blue), 75%-FAT/110 mm (green) and 50%-FAT/110 mm (orange) **(A/B upper)**. The tumor is shown in yellow. In case 3, the neurophysiology-driven approach resulted in an exclusive fiber tract anterior to the lesion in contrast to the standard approaches. In case 4, our approach resulted in an additional fiber tract cranial to the lesion compared to the 75%-FAT approach. Location of tracts were confirmed intraoperatively by direct cortical electrical stimulation **(A/B lower)**.

**FIGURE 6:**
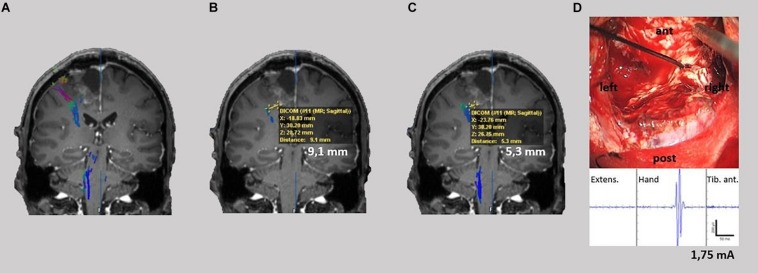
TMS-based DTI fiber tracking of patient no. 5. Comparison between tracts resulting from FA and FL parameter selection with our neurophysiology-driven approach **(A)**, 75%-FAT/110 mm **(B)** and 50%-FAT/110 mm **(C)** with shortest distance of the determined tracts to the resection cave. Lowest stimulation intensity leading to a motor response during intraoperatively subcortical stimulation **(D)**.

Finally, in nine patients DCS and in four patients SCS were performed intraoperatively. Stimulation results were photo-documented and postoperative compared with the DTI-based determined fiber tracts due to anatomical landmarks and postoperative MRI scans (Figures 5, 6). In the cases of patients with SCS we analyzed the minimum distance of the resection cavity from the determined fiber tracts in postoperative MRI and compared it with intraoperative SCS intensities (Figure 6).

### Statistics

All statistical tests were performed using MATLAB (MathWorks, Inc., Natick, MA, United States). We analyzed the goodness-of-fit (i.e., the coefficient of determination *r*^2^) of the mathematical model for the multivariate dependency of FA, FL, and NoF as well as the Pearson’s correlation coefficient *r* for the relationship between NoF and MEP amplitudes. Data are shown as the mean ± standard deviation (SD), minimum, and maximum values.

## Results

### Mathematical Model for the Relationship Between FA, FL, and NoF and Neurophysiology-Driven DTI Parameter Selection

Automated neurophysiology-driven selection of optimized DTI parameters was possible in all patients. Fitting of the relationship between FA and FL threshold as well as NoF resulted in S-shaped curves. There was a good goodness-of-fit for the mathematical model in 80% (8/10) of the patients (*r*^2^ = 0.68 ± 0.13; range: 0.59–0.97; *n* = 8). In two cases fitting the mathematical model was insufficient (Table 1). Nevertheless, automated parameter selection resulted in a high correlation between DTI-FT and nTMS results (*r* = 0.73 ± 0.16; range: 0.38–0.93; *n* = 8).

### Comparison to the Standard Approach

#### Patients With a Sufficient Goodness-of Fit

Relating the DTI parameters of the neurophysiology-driven approach to the values of the standard approaches, we detected FL thresholds <110 mm in 50% (4/8) of the patients with a sufficient goodness-of fit. Notably, on the group level, correlation results led to FL thresholds around 90 mm (Figure 4). However, individual FL thresholds in our patients fluctuated between 85 and 170 mm.

The FA threshold was higher than the 75%- and 50%-FAT in 25% (2/8) of patients. In 50% (4/8) of cases, the mathematical model resulted in a FA threshold between the 75%- and 50%-FAT. Moreover, in 25% (2/8) the mathematical model resulted in values lower than its corresponding standard FAT-values (Table 2). In 37.5% of the patients (3/8), the analysis resulted in a NoF higher than the 75%-FAT and lower than the 50%-FAT values and in 25% (2/8) in a NoF lower than the 75%- and 50%-FAT.

The graphical comparison of the fiber tracts did not show any additional information (e.g., aberrant fibers) other than an increased or decreased tract diameter in 50% of the cases (patients no. 1, 2, 7, and 8). However, in 50% (patients no. 3, 4, 5, and 6) our approach changed the detected tract anatomy. Here, the neurophysiology-driven approach helped to clarify the exact tract location (Figure 5A) or detect an additional functional tract which was not detected by the standard approach (Figure 5B), especially in cortical areas. This could be confirmed with DCS and/or SCS in all four cases. However, in one of these (patient no. 5), neither the standard nor the neurophysiology-driven approach seems to be appropriate for DTI-FT (Figure 6). On the one hand, the incongruity of the distance between determined tracts of the 75%-FAT approach and the resection cave and the very low intraoperative stimulation intensities (1.75 mA), which could evoke motor responses, is probably caused by a tract underestimation. On the other hand, our approach seems to overestimate tracts.

#### Patients With a Limited Goodness-of Fit

**FIGURE 7:**
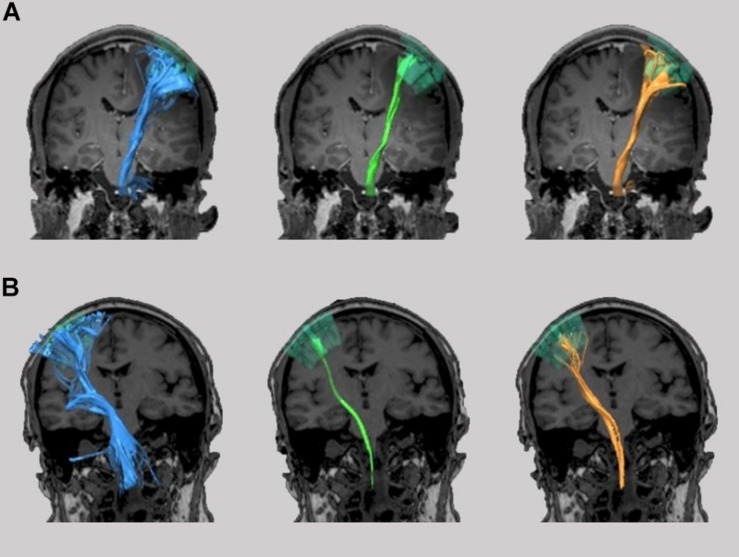
TMS-based DTI fiber tracking of patient no. 9 **(A)** and 10 **(B)**. Comparison between tracts resulting from FA and FL parameter selection with our neurophysiology-driven approach (blue), 75%-FAT/110 mm (green), and 50%-FAT/110 mm (orange).

In the analysis of patients with a limited goodness-of fit, we detected FL thresholds <110 mm and >110 mm each in 50% (1/2) of the patients. The FA threshold was higher than the 75%- and 50%-FAT in both cases. This resulted in a NoF higher than the 75%-FAT and the 50%-FAT approach in 50% (1/2; patient no. 10) and in a NoF lower than the 75%-FAT and the 50%-FAT approach in 50% (1/2, patient no. 9) (Figure 7).

## Discussion

There is an increasing interest in the concept of combining nTMS and DTI-FT during preoperative neurosurgical planning. In this context, the aim of our study was to investigate a neurophysiology-driven approach to FA and FL threshold selection for DTI-FT of the CST and to compare it to standard parameter selection.

In regard to this goal, we demonstrate the feasibility of a completely user-independent mathematical modeling of the correlation between the NoF detected by the DTI-FT and the MEP amplitude of the nTMS, with a sufficient goodness-of-fit in 80% of the patients and a high correlation between DTI-FT and nTMS results. The individual FA thresholds differ from the postulated 75%-FAT in all patients. Importantly, in 50% of the patients the neurophysiology-driven adaptation of the DTI thresholds changes the detected tract anatomy significantly in the sense of clarifying the exact tract location or detecting additional functional tracts, which are not detected by the standard method. Notably, the presented neurophysiology-driven approach is superior in cases where standard nTMS-DTI has difficulties to detect the cortical course of fiber tracts, e.g., in a situation with an extended edema (e.g., metastases) or in a superficial but subcortical lesion. However, in 12.5% of the patients neither the standard nor our approach seems to be appropriate for DTI-FT. An improvement or modification of the mathematical model might help to overcome this drawback.

In the past, several studies have shown that ROI seeding based on nTMS data resulted not only in more specific tracts with less aberrant fibers, but reduced the variability between examiners compared to the seeding approach using anatomical landmarks (Frey et al., 2012; Krieg et al., 2012; Weiss Lucas et al., 2017). In order to improve the threshold selection, previous standardization attempts suggested a FL threshold of 110 mm and the implementation of a 100%-FAT criterium. The latter described the FA threshold at which only a thin fiber tract was visualized and all other FA values were described in relation to this 100%-FAT value as e.g., 75%-, 50%- or 25%-FAT (Frey et al., 2012; Weiss et al., 2015; Rosenstock et al., 2017; Weiss Lucas et al., 2017). The concept of performing fiber tracking with a FL threshold of 110 mm and a 75%-FAT resulted in a more objective tractography than the previous methods (Frey et al., 2012). Nevertheless, this remains an arbitrary value selection which neither takes the interindividual patient characteristics into account nor the different types of fiber tracts (e.g., CST or language pathways). Furthermore, there currently exists contention concerning the ambiguity of the standardized values (i.e., 75%-, 50%- or 25%-FAT) to be used (Sollmann et al., 2016; Weiss Lucas et al., 2017). The proposed neurophysiology-driven approach for FA and FL threshold selection not only chooses the threshold parameters in a completely user-independent manner, but also considers the differences between patients. In line with this, the calculated FA and FL thresholds in the present study showed variations in both directions compared to the 75%- and 50%-FAT and a FL threshold of 110 mm. The differences of our thresholds compared to the standard values seem to represent these individual differences between patients.

In the final comparison of fiber tracking with the nTMS-ROI as cortical region-of-interest, our automatically collected thresholds resulted in a higher NoF in 75% of the patients compared to the arbitrary thresholds of 75%-FAT and FL threshold of 110 mm. Considering the known underestimation of fibers in the cortex region in DTI algorithms (Kinoshita et al., 2005; Farquharson et al., 2013), our approach may improve the sensitivity of DTI fiber tracking in this region. Furthermore, the NoF may be challenged in cases with tumor edema. It has been shown that edema can lead to significant changes in the measured FA values and thus to a misinterpretation of the determined pathways (Kinoshita et al., 2005). In case 3, a pronounced perifocal tumor edema resulted in an apparently excessive number of pathways with the standard DTI-thresholding, which was not confirmed intraoperatively. Our suggested neurophysiology-driven approach, however, helped to filter these findings such that they fit to the intraoperative situation. Thus, we hypothesize that the proposed introduction of an additional information in the tracking process (i.e., MEP amplitudes) has the potential to falsify aberrant tracts and to reduce the detection of false positive or false negative tracts. This is very important in brain tumor surgery, as stopping surgery to preserve false positive fibers might result in a subtotal resection which has a negative impact on over-all survival of the patient (Brown et al., 2016). On the other side, continuation of surgery due to false-negative tractography will cause postoperative deficits which will affect patient’s quality of life and will have a negative impact on his survival as well (Rahman et al., 2017). Thus, precise tractography is relevant to neurosurgical procedures within the dual aims of a preferably comprehensive extent of resection and preservation of eloquent brain areas.

### Limitations of the Study

Alongside other DTI tractographies, the suggested neurophysiology-driven approach still lacks ground truth information and may only reduce, but not avoid, false positive or false negative tracts, e.g., due to the crossing and “kissing fiber” problem (Jbabdi and Johansen-Berg, 2011; Essayed et al., 2017). Therefore, an exact conclusion, whether and in which situations our method is superior to previous nTMS based approaches is not possible with the present study. In particular, it remains unclear why in two cases the fitting of the neurophysiology approach was insufficient. However, there are several factors which could have influenced our results. First, perilesional edema could have led to a reduction of FA values in this region and thus to significantly broader fiber tracts (Kinoshita et al., 2005). Second, the accuracy of our neurophysiological model may be inaccurate in cases suffering from pre-operative paresis. However, it should be mentioned that in three out of four patients of our study, who suffered from an upper limb paresis preoperatively, the model exhibited a good goodness-of-fit. Notably, we have seen a superiority of our method in two of these cases, since the localization of the tract was apparently more reliable. In addition to the limitations mentioned above, the fitting model takes into account only the factors FA- and FL-threshold, but no further aspects like the angulation threshold or the apparent diffusion coefficient. Therefore, an improvement of the applied mathematical model could be achieved by integrating these factors. Furthermore, a systematic investigation of the applied model in healthy subjects, a larger patient cohort and other white matter tracts than the CST is indicated to validate our results. Furthermore, a more sophisticated (intraoperative) validation of the calculated fiber tracts should be performed with SCS and DCS, e.g., in combination with neuronavigation or intraoperative imaging.

## Conclusion

This present study is the first to introduce a standardized and nTMS-based threshold selection for DTI-FT. Implementation and further exploration of this approach could overcome some of the current limitations of concurrent DTI-TMS approaches and ultimately result in an improvement of neurosurgical planning.

## Data Availability Statement

The datasets generated for this study are available on request to the corresponding author.

## Ethics Statement

The studies involving human participants were reviewed and approved by the local ethics committee, Eberhard Karls Universität Tübingen. Written informed consent for participation was not required for this study in accordance with the national legislation and the institutional requirements.

## Author Contributions

KMa contributed to the acquisition, analysis, and interpretation of data and writing of the first draft. LT, MTL, ML, KMo, BB, UE, AG, and MT contributed to the data acquisition, interpretation of data, and the review and critique of the final manuscript. GN was responsible for the conception, design, data acquisition, analysis, and interpretation as well as the review and critique of the manuscript.

## Conflict of Interest

The authors declare that the research was conducted in the absence of any commercial or financial relationships that could be construed as a potential conflict of interest.

## Abbreviations

APB: abductor pollicis brevis
CST: corticospinal tract
DCS: direct cortical stimulation
DTI: diffusion tensor imaging
DTI-FT: diffusion tensor imaging fiber tracking
EDC: extensor digitorum communis
EMG: electromyography
FA: fractional anisotropy
FAT: fractional anisotropy threshold
FD: flexor digitorum
FL: fiber length
HARDI: high angular resolution diffusion imaging
MEP: motor evoked potential
MRI: magnetic resonance imaging
NoF: number of fibers
nTMS: navigated transcranial magnetic stimulation
rMT: resting motor threshold
ROI: region of interest
SCS: subcortical stimulation

## References

[B1] Assaf Y. Pasternak O. (2008). Diffusion tensor imaging (DTI)-based white matter mapping in brain research: a review. *J. Mol. Neurosci.* 34 51–61. 10.1007/s12031-007-0029-0 18157658

[B2] Basser P. J. Pajevic S. Pierpaoli C. Duda J. Aldroubi A. (2000). In vivo fiber tractography using DT-MRI data. *Magn. Reson. Med.* 44 625–632. 10.1002/1522-2594(200010)44:4<625::aid-mrm17>3.0.co;2-o 11025519

[B3] Brown T. J. Brennan M. C. Li M. Church E. W. Brandmeir N. J. Rakszawski K. L. (2016). Association of the extent of resection with survival in glioblastoma a systematic review and meta-analysis. *JAMA Oncol.* 2 1460–1469. 10.1001/jamaoncol.2016.1373 27310651 PMC6438173

[B4] Chung H. W. Chou M. C. Chen C. Y. (2011). Principles and limitations of computational algorithms in clinical diffusion tensor MR tractography. *Am. J. Neuroradiol.* 32 3–13. 10.3174/ajnr.A2041 20299436 PMC7964942

[B5] Clark C. A. Barrick T. R. Murphy M. M. Bell B. A. (2003). White matter fiber tracking in patients with space-occupying lesions of the brain: a new technique for neurosurgical planning? *Neuroimage* 20 1601–1608. 10.1016/j.neuroimage.2003.07.022 14642471

[B6] Essayed W. I. Zhang F. Unadkat P. Cosgrove G. R. Golby A. J. O’Donnell L. J. (2017). White matter tractography for neurosurgical planning: a topography-based review of the current state of the art. *NeuroImage Clin.* 15 659–672. 10.1016/j.nicl.2017.06.011 28664037 PMC5480983

[B7] Farquharson S. Tournier J. D. Calamante F. (2013). White matter fiber tractography: why we need to move beyond DTI. *J. Neurosurg.* 118 1367–1377. 10.3171/2013.2.JNS121294 23540269

[B8] Frey D. Strack V. Wiener E. Jussen D. Vajkoczy P. Picht T. (2012). A new approach for corticospinal tract reconstruction based on navigated transcranial stimulation and standardized fractional anisotropy values. *Neuroimage* 62 1600–1609. 10.1016/j.neuroimage.2012.05.059 22659445

[B9] Hervey-Jumper S. L. Berger M. S. (2016). Maximizing safe resection of low- and high-grade glioma. *J. Neurooncol.* 130 269–282. 10.1007/s11060-016-2110-4 27174197

[B10] Jakola A. S. Myrmel K. S. Kloster R. Torp S. H. Lindal S. Unsgård G. (2012). Comparison of a strategy favoring early surgical resection vs a strategy favoring watchful waiting in low-grade gliomas. *JAMAJ. Am. Med. Assoc.* 308 1881–1888. 10.1001/jama.2012.12807 23099483

[B11] Jbabdi S. Johansen-Berg H. (2011). Tractography: where do we go from here? *Brain Connect* 1 169–183. 10.1089/brain.2011.0033 22433046 PMC3677805

[B12] Kamada K. Sawamura Y. Takeuchi F. Kawaguchi H. Kuriki S. Todo T. (2005). Functional identification of the primary motor area by corticospinal tractography. *Neurosurgery* 56 98–109. 10.1227/01.NEU.0000144311.88383.EF 15799797

[B13] Kinoshita M. Yamada K. Hashimoto N. Kato A. Izumoto S. Baba T. (2005). Fiber-tracking does not accurately estimate size of fiber bundle in pathological condition: initial neurosurgical experience using neuronavigation and subcortical white matter stimulation. *Neuroimage* 25 424–429. 10.1016/j.neuroimage.2004.07.076 15784421

[B14] Krieg S. M. Buchmann N. H. Gempt J. Shiban E. Meyer B. Ringel F. (2012). Diffusion tensor imaging fiber tracking using navigated brain stimulation - A feasibility study. *Acta Neurochir.* 154 555–563. 10.1007/s00701-011-1255-3 22270529

[B15] Maier-Hein K. H. (2017). The challenge of mapping the human connectome based on diffusion tractography. *Nat. Commun.* 8 1–13. 10.1038/s41467-017-01285 29116093 PMC5677006

[B16] Nimsky C. Ganslandt O. Merhof D. Sorensen A. G. Fahlbusch R. (2006). Intraoperative visualization of the pyramidal tract by diffusion-tensor-imaging-based fiber tracking. *Neuroimage* 30 1219–1229. 10.1016/j.neuroimage.2005.11.001 16364659

[B17] Ottenhausen M. Krieg S. M. Meyer B. Ringel F. (2015). Functional preoperative and intraoperative mapping and monitoring: increasing safety and efficacy in glioma surgery. *Neurosurg. Focus* 38:E3. 10.3171/2014.10.FOCUS14611 25552283

[B18] Raffa G. Conti A. Scibilia A. Sindorio C. Quattropani M. C. Visocchi M. (2017). Functional reconstruction of motor and language pathways based on navigated transcranial magnetic stimulation and DTI fiber tracking for the preoperative planning of low grade glioma surgery: a new tool for preservation and restoration of eloquent network. *Acta Neurochir., Suppl.* 124 251–261. 10.1007/978-3-319-39546-3_37 28120081

[B19] Rahman M. Abbatematteo J. De Leo E. K. Kubilis P. S. Vaziri S. Bova F. (2017). The effects of new or worsened postoperative neurological deficits on survival of patients with glioblastoma. *J. Neurosurg.* 127 123–131. 10.3171/2016.7.JNS16396 27689459

[B20] Rosenstock T. Giampiccolo D. Schneider H. Runge S. J. Bährend I. Vajkoczy P. (2017). Specific DTI seeding and diffusivity-analysis improve the quality and prognostic value of TMS-based deterministic DTI of the pyramidal tract. *NeuroImage Clin.* 16 276–285. 10.1016/j.nicl.2017.08.010 28840099 PMC5560117

[B21] Sollmann N. Negwer C. Ille S. Maurer S. Hauck T. Kirschke J. S. (2016). Feasibility of nTMS-based DTI fiber tracking of language pathways in neurosurgical patients using a fractional anisotropy threshold. *J. Neurosci. Methods* 267 45–54. 10.1016/j.jneumeth.2016.04.002 27059128

[B22] Tarapore P. E. Tate M. C. Findlay A. M. Honma S. M. Mizuiri D. Berger M. S. (2012). Preoperative multimodal motor mapping: a comparison of magnetoencephalography imaging, navigated transcranial magnetic stimulation, and direct cortical stimulation. *J. Neurosurg.* 117 354–362. 10.3171/2012.5.JNS112124 22702484 PMC4060619

[B23] Weiss C. Tursunova I. Neuschmelting V. Lockau H. Nettekoven C. Oros-Peusquens A. M. (2015). Improved nTMS- and DTI-derived CST tractography through anatomical ROI seeding on anterior pontine level compared to internal capsule. *NeuroImage Clin.* 7 424–437. 10.1016/j.nicl.2015.01.006 25685709 PMC4314616

[B24] Weiss Lucas C. Tursunova I. Neuschmelting V. Nettekoven C. Oros-Peusquens A. M. Stoffels G. (2017). Functional MRI vs. navigated TMS to optimize M1 seed volume delineation for DTI tractography. A prospective study in patients with brain tumours adjacent to the corticospinal tract. *NeuroImage Clin.* 13 297–309. 10.1016/j.nicl.2016.11.022 28050345 PMC5192048

